# Weight and height separated provide better understanding than BMI on the risk of revision after total knee arthroplasty: report of 107,228 primary total knee arthroplasties from the Swedish Knee Arthroplasty Register 2009–2017

**DOI:** 10.1080/17453674.2019.1688006

**Published:** 2019-11-08

**Authors:** Erdem A Sezgin, Annette W-Dahl, Lars Lidgren, Otto Robertsson

**Affiliations:** a Gazi University, Faculty of Medicine, Department of Orthopedics and Traumatology, Ankara, Turkey;; b Lund University, Faculty of Medicine, Department of Clinical Sciences Lund, Orthopedics, Lund, Sweden;; c The Swedish Knee Arthroplasty Register, Lund, Sweden

## Abstract

Background and purpose — Obesity defined as increased BMI is commonly associated with higher revision rates following total knee arthroplasty (TKA). We examined the effect of BMI on the rate of revision after TKA, for both infection and other reasons, and analyzed weight and height separately to provide better understanding of the risk profile.

Patients and methods — The Swedish national knee arthroplasty register was used to identify 107,228 patients operated with primary TKA for osteoarthritis between 2009 and 2017. Cox proportional hazards regression was used to calculate hazard ratios (HRs) with 95% confidence intervals (CIs) for BMI (categories: < 18.5, 18.5–24.9, 25–29.9, 30–34.9, 35–39.9, ≥ 40), weight (categories: < 65, 65–89, 90–114, ≥ 115 kg) and height (categories: < 160, 160–179, ≥ 180 cm

Results — There were 2,503 revisions in the follow-up period; 1,036 for infection and 1,467 for other reasons. Higher BMI and weight categories were associated with a similar and statistically significantly increased risk of revision for all causes and for infection. The risk of revision for infection was almost twice in the highest BMI and highest weight group: HR = 3.4 (CI 2.3–4.7) and HR = 3.1 (CI 2.5–3.9) respectively. For BMI and weight categories there was no statistically significant association between revision for other reasons than infection, contrary to the tallest height category where it was statistically significant (HR = 1.3 [CI 1.1–1.5]).

Interpretation — BMI, weight, and height may be associated with different types of risks for revision following TKA.

Patients having a total knee arthroplasty (TKA) have some modifiable risk factors that may affect surgical outcome. Obesity has reached epidemic levels mainly in the western countries and is being considered as a public health crisis (Gonzalez Della Valle et al. 2012, George et al. 2017). Obesity is considered a risk factor for both developing knee osteoarthritis (OA) and progression requiring TKA surgery at younger age (Changulani et al. 2008, Singer et al. 2018). Moreover, many studies have shown that morbid obesity is associated with increased risk of revision following TKA (Springer et al. 2013, Roche et al. 2018, Tohidi et al. 2018, Boyce et al. 2019). However, most of the studies report overall risk for revision without detailing reasons for revision. The effect of obesity on revision besides an increased risk for periprosthetic joint infection (PJI) has not been thoroughly discussed in the literature (D’Apuzzo et al. 2015, Wagner et al. 2016, Jung et al. 2017, Roche et al. 2018, Boyce et al. 2019). There might be a complex relation between weight and height, and TKA outcome. Only 1 study has shown that weight and height effect the risk of revision differently when analyzed as independent variables (Christensen et al. 2018).

Therefore, this nationwide register-based study in Sweden evaluated the effect of BMI on rate of revision for PJI as well as reasons for revision other than PJI in patients operated for OA with TKA, and analyzed weight and height separately to provide better understanding of risk of revision.

## Patients and methods

Patients who had undergone primary TKA for OA between January 1, 2009 and December 31, 2017 were identified in the Swedish Knee Arthroplasty Register (SKAR). The SKAR, which started in 1975, is the world’s first national arthroplasty register and has high coverage and completeness (SKAR 2018).

BMI, weight, and height at the time of surgery were identified from the SKAR as well as age and sex. TKAs lacking BMI data were excluded. The World Health Organization (WHO) classification was used to classify BMI into 6 categories: underweight (BMI < 18.5), normal weight (BMI 18.5–24.9), overweight (BMI 25–29.9), obese class I (BMI 30–34.9), obese class II (BMI 35–39.9), and obese class III (BMI ≥ 40). Weight and height were analyzed as both continuous and categorical variables. Categories for weight and height were defined arbitrarily: < 65, 65–89, 90–114, ≥ 115 kg; and < 160, 160–179, ≥ 180 cm.

The outcome measures were revision for all reasons, revision for infection or suspected infection, and revision for reasons other than infection. In the SKAR, revisions are defined as a new operation in which 1 or more of the components are changed, removed, or added (including arthrodesis and amputation). Patients included in the study had a follow-up until death or December 31, 2017.

### Statistics

Adjusted hazard ratios (HRs) were obtained for each 6 BMI categories using Cox proportional hazards regressions with 3 separate endpoints: revision for all reasons, revision for infection, and revision for reasons other than infection. Adjustments were made for differences in age and sex at the time of operation. Similarly, Cox proportional hazards regression was used to obtain adjusted HRs for weight and height as both continuous and categorical variables using the aforementioned 3 outcomes as endpoints. Reference categories were determined for BMI, weight, and height as 18.5–24.9, 65–89 kg, and 160–179 cm respectively. Adjustments were made for differences in age, sex, weight, and height at the time of surgery.

Results are reported as HRs with 95% confidence intervals (CIs). P-values of < 0.05 were considered to be statistically significant. Statistical analyses were carried out using Stata version 15 (StataCorp, College Station, TX, USA).

### Ethics, funding, and potential conflicts of interest

The data gathering from the Swedish Knee Arthroplasty Register was approved by the Ethics Board of Lund University (LU20-02). The authors received no funding for this work. No conflicts of interest were declared.

## Results

From the SKAR database 108,623 TKAs were identified that met the initial eligibility criteria. 1,395 TKAs with missing data were excluded and 107,228 TKAs were included in the study cohort. For the BMI categories, 0.2% of TKAs were underweight, 18% normal weight, 43% overweight, 28% in obese class I, 8.6% in obese class II, and finally 1.9% were in obese class III. For weight and height categories 8.3% were in < 65 kg, 57% in 65–89 kg, 31% in 90–114 kg, 3.8% in ≥ 115 kg, and 46% in < 160 cm, 68.0% in 160–179 cm, 19% in ≥180 cm. There were 2,503 (2.3%) revisions over the follow-up period, 1,036 being for infection and 1,467 for reasons other than infection.

### BMI and revision

After Cox proportional hazard regression, considering revision for all reasons, obesity was found to be associated with higher risk starting from obese class I (Table 1). Revision for infection also demonstrated a similar increase of risk starting from obese class I. However, no such trend between obesity and revision rates for reasons other than infection was observed except for the increase in obese class I.

**Table 1.** Adjusted hazard ratios (HR) for different outcomes according to the 6 BMI categories

BMI	Revision (all causes) HR (CI)	Revision for infection HR (CI)	Revision for reasons other than infection HR (CI)
< 18.5	0.3 (0.1–2.1)	0.8 (0.1–5.7)	Not available
18.5–24.9	Reference		
25–29.9	1.1 (0.9–1.2)	1.1 (0.9–1.3)	1.1 (0.9–1.3)
30–34.9	1.3 (1.1–1.5)	1.5 (1.2–1.8)	1.2 (1.01–1.4)
35–39.9	1.3 (1.1–1.5)	2.1 (1.7–2.7)	0.9 (0.7–1.1)
≥ 40	1.6 (1.2–2)	3.3 (2.3–4.7)	0.9 (0.6–1.3)

CI = 95% confidence interval

### Weight and revision

Like BMI, weight also demonstrated an association with risk of revision and revision for infection, as both a continuous and a categorical variable. However, no statistically significant difference was found between the weight groups and revision for reasons other than infection (Table 2).

**Table 2.** Adjusted hazard ratios (HR) for different outcomes according to weight and height as continuous and categorical variables

Parameter	Revision (all causes) HR (CI)	Revision for infection HR (CI)	Revision for reasons other than infection HR (CI)
Weight (kg)			
Continuous	1.01 (1.01–1.01)	1.02 (1.02–1.03)	1 (1–1)
< 65	0.9 (0.7–1.02)	0.7 (0.5–1)	0.9 (0.8–1.2)
65–89	Reference		
90–114	1.2 (1.1–1.3)	1.4 (1.2–1.6)	1.1 (0.95–1.2)
≥ 115	1.8 (1.5–2.1)	3.1 (2.5–3.9)	1.1 (0.8–1.4)
Height (cm)			
Continuous	1.01 (1.00–1.02)	0.99 (0.98–1.00)	1.02 (1.02–1.03)
< 160	0.9 (0.8–1.1)	1.3 (1.02–1.6)	0.8 (0.7–1)
160–179	Reference		
≥ 180	1.1 (1–1.3)	1 (0.8–1.2)	1.3 (1.1–1.5)

CI = 95% confidence interval

### Height and revision

Although height as a continuous variable had a statistically significant association with all revisions, there were no statistically significant differences between height categories and revision. In addition, medium and tall height categories did not show statistically significant association with revision for infection; however, there was increased risk in the shortest height category. Contrary to other endpoints, we found that the tallest height category and height as a continuous variable were associated with higher risk of revision for reasons other than infection (Table 2).

## Discussion

We showed that obesity was associated with overall risk of revision and revision for infection, but the same relationship could not be shown for revision for reasons other than infection. The 2 heaviest groups and weight as a continuous variable demonstrated a risk similar to that of obesity whereas tallest height category and 1 cm increase in height appeared to be associated with higher risk of revision for reasons other than PJI. In addition, shorter patients appeared to have increased risk of revision for PJI but there is no basis in our study and the literature to comment on this finding.

The results were based on 107,228 patients from the SKAR, which has high completeness and response rate and covers the whole nation. However, we acknowledge several limitations to our study. First, despite overweight and obesity increase in the Swedish population, the percentages are still lower than in many other countries and, further, the average height of the Swedish population is higher than the world average (Hanson et al. 2009). Second, it may be argued that weight and height are naturally dependent variables. However, it should be noted that the focus of this study was not to introduce height and weight as alternative predictors of outcome; such analysis has been done recently by Christensen et al. (2018) using a similar statistical method. Moreover, as height is not a modifiable parameter, clinical relevance of height is questionable and these results can only be used as a base for further speculations on mechanical effects of height. Finally, we would like to note that the study groups were arbitrarily defined, as in other studies, due to the lack of defined weight and height categories considering the risk of revision. While deciding on the groups, we tried to portray TKA patient profiles according to orthopedic surgeons’ (EAS, LL, OR) clinical experiences. Normal and overweight BMI categories totaled approximately 70% of patients and the 65–89 kg category totaled 60% in our study. Similarly, there were 30% in both the 90–114 kg category and obese class 1. There were 4% in the heaviest category and 10% in obese class 3. Also, for height, approximately 70% were in the normal, 10% in the short, and 20% in the tall group. We acknowledge that arbitrary categories and consequent sample sizes have a considerable effect on statistical analysis, especially for height, where this parameter showed a correlation with higher risk of overall revision as a continuous variable; however, there was no correlation between height categories for the same endpoint.

By definition BMI is used to determine healthy weight and its ability to reflect body habitus is limited. Therefore, the individual variables, height and weight, used for calculation of BMI have recently drawn attention and are also suggested to be valuable as alternative predictors of risk (Lübbeke et al. 2016, Christensen et al. 2018, Gøttsche et al. 2019). Being the numerator in the calculation, weight has a positive correlation with BMI contrary to height, which is the denominator. Therefore, weight and height can have an effect on their own and not necessarily in combination. This concept has hitherto only been discussed by Christensen et al. (2018). Their single-center study consisting of more than 20,000 consecutive TKAs performed between 1985 and 2012 showed that increasing BMI, body weight, and body surface area was associated with an increased risk of infection, although height demonstrated no correlation in multivariate models. Instead, they showed that each 11 cm increase in height resulted in 14% increased risk of revision for mechanical failure. Our study supports this concept, by pointing out that height has a different effect on rate of revision for reasons other than PJI compared with weight and BMI. It can be translated as suggesting that a tall and obese individual would be more at risk for mechanical failure than a short and obese one, while both have increased risk of revision for infection after TKA. The reason behind this might be the increased lever arm, which results in higher moment of force above and below prostheses in taller patients, thus creating unfavorable mechanical loading that can lead to premature wear and loosening (Christensen et al. 2018). In addition, considering taller patients are not expected to adopt a more sedentary lifestyle like obese patients tend to do, it is plausible that height has a possible effect on mechanical complications after TKA which differs from that of BMI and weight (McClung et al. 2000, Amin et al. 2006, Overgaard et al. 2019). Weight, on the other hand, has been discussed several times in the literature. Lübbeke et al. (2016) analyzed the effect of weight on PJI rates in arthroplasty and suggested that both 35 kg/m^2^ and 100 kg can be regarded as thresholds for a significant 2-fold increase in PJI. Gøttsche et al. (2019) also studied effects of weight on implant survival but found no correlation between weight and revision for aseptic loosening or infection. Despite being a Danish nationwide registry study with almost 70,000 TKAs, there were only 173 (0.3%) revisions due to infection, which may explain the lack of correlation or indicate possible under-reporting in their study.

In conclusion, our results could aid risk assessment and consideration of possible precautions to reduce failure but not patient selection for TKA. As height is not a modifiable factor, taller patients could be informed about the possible value of decreasing the load on the TKA by losing weight and modifying high-impact activity.
